# Research on Interrupted Sampling Repeater Jamming Performance Based on Joint Frequency Shift/Phase Modulation

**DOI:** 10.3390/s23052812

**Published:** 2023-03-04

**Authors:** Jie Xiao, Xizhang Wei, Jia Sun

**Affiliations:** School of Electronics and Communication Engineering, Sun Yat-sen University, Shenzhen 518000, China

**Keywords:** electronic countermeasures, ISRJ, frequency shift modulation, phase modulation

## Abstract

Interrupted sampling repeater jamming (ISRJ) is a classic active coherent jamming. Due to its structural limitations, it has inherent defects such as a discontinuous time–frequency (TF) distribution, strong distribution regularity of pulse compression results, limited jamming amplitude, and strong false targets lagging behind the real target. These defects have not been fully resolved yet due to the limitation of the theoretical analysis system. Based on the analysis of the influence factors of ISRJ on the interference performance for linear-frequency-modulated (LFM) and phase-coded signals, this paper proposes an improved ISRJ method based on the joint subsection frequency shift and two-phase modulation. The coherent superposition of jamming signals at different positions for LFM signals is achieved by controlling the frequency shift matrix and the phase modulation parameters to form a strong pre-lead false target or multiple positions and ranges of blanket jamming areas. For the phase-coded signal, the pre-lead false targets are generated through code prediction and the two-phase modulation of the code sequence, resulting in similar noise interference. The simulation results show that this method can overcome the inherent defects of ISRJ.

## 1. Introduction

The pulse compression (PC) radar can obtain higher coherent processing gain after matched filtering, which provides effective robustness against jamming signals that do not match the radar waveform. In recent years, thanks to the development of digital radio frequency memory (DRFM) equipment [1,2], interrupted sampling repeater jamming (ISRJ) has been a common jamming style in the field of electronic countermeasures [3,4]. Based on DRFM and “under-sampling” technology, ISRJ periodically slices and circularly forwards intercepted radar signals. ISRJ has the advantages of fast response speed, high isolation, and easy engineering implementation, and can achieve both deception and blanket jamming.

However, due to the limitations of its inherent structure, there are obvious differences in the interference effect for different pulse compression signals. The main drawbacks of ISRJ for linear-frequency-modulated (LFM) and phase-coded signals are expressed in [3,4,5,6,7,8,9,10,11] as follows:

- Its time–frequency (TF) distribution is the same within an interrupted sampling period, and the distribution characteristics are discontinuous.
- The pulse compression results have a strong distribution regularity for different signals. A set of false target strings are generated for the LFM signal, and the multi-order false target is centered on the zero-order false target, showing a symmetrical attenuation distribution on both sides. The amplitude decays rapidly with the increase in the order. There is only one lagging false target for the phase-coded signal.
- The jamming amplitude is limited and proportional to the duty cycle. The attenuation speed of multi-order false target amplitude is proportional to the duty cycle for the LFM signals.
- The zero-order false target lags behind the real target by at least one interrupted sampling pulse width. The smaller the interrupted sampling pulse width, the smaller the distance between the zero-order false target and the real target, and vice versa. Therefore, when the interrupted sampling pulse width is small, the detection probability of the real target can be enhanced in one range cell. When the interrupted sampling pulse width is large, the lag time of the zero-order target is longer, which may expose the real target.

In recent years, time–frequency analysis [12], filter design [13,14], waveform optimization design [15,16], and interference cancellation [17] can effectively counteract ISRJ. Therefore, to solve the drawback of ISRJ and effectively counteract the various new anti-interference methods, improved ISRJ methods are urgently needed. The modulation method for ISRJ has become a research hotspot, including time–domain modulation, frequency–domain modulation, and phase modulation.

For LFM signals, modulation methods for different domains have been proposed to break the strong distribution regularity of ISRJ. These effectively control the amplitude and interval of the false target and cause the jamming difficulty to be recognized by enemy radars [18,19,20,21,22,23,24,25,26]. Time–domain modulation mainly includes non-periodic interrupted sampling modulation [18], periodic cyclic coded intermittent sampling modulation [19], interrupted-sampling and non-uniform periodic repeater jamming (ISNPRJ) [20], and multi-waveform modulation [21]. It can produce controllable densely distributed false targets and weaken the distribution regularity of false targets. However, these time–domain modulation methods cannot solve the problem of false target lag. Frequency–domain modulation can control the position of a false target by controlling the frequency shift parameters, forming blanket jamming or deceptive jamming. However, due to the limited jamming power, the interference amplitude and the interference effect are poor [22,23]. Phase modulation can generate noise-like interference. The number and density of effective false targets can be changed by adjusting the two-phase and multi-phase parameters. Multiple jamming areas with controllable positions and ranges can be generated to expand the interference range and weaken the regularity of interference distribution. However, the disadvantage is the low interference amplitude [24,25,26].

In recent years, there have been few studies on ISRJ modulation methods for phase-coded signals [27,28,29,30,31]. The interference effect of ISRJ is that only a high-fidelity false target with hysteresis is generated [27,28]. The ISRJ based on pseudo-random sequence phase modulation forms dense false targets. However, their amplitudes decrease, and their positions and amplitudes are uncontrollable [29]. Based on the characteristics of group delay, it is gathered that frequency shift modulation of ISRJ cannot form pre-lead false targets for phase-coded signals. The range of leading and lagging false targets is controlled by predicting and adjusting code sequences [30,31]. Frequency-domain modulation causes matched filter detuning for phase-coded signal, which leads to a sharp drop in the jamming amplitude. The adjustment of the phase modulation parameters through code sequence prediction can form a pre-lead false target.

A single modulation method cannot simultaneously solve the multiple challenges faced by ISRJ. Considering the advantages of the ISRJ modulation method for different radar signals, the interference effect of the improved ISRJ methods is further analyzed in [32,33,34]. Two-time sequence parameter design strategies of time-delay superposition jamming are proposed, which can improve the processing gain by adjusting the phase of time-delay superposition samples [32]. Multicarrier modulation and retransmission jamming based on interrupted sampling is proposed [33]. It has the characteristics of high jamming mode complexity, strong coherence, a large number of false targets, and a wide distribution range. Appling Doppler frequency shift and non-uniform forwarding to ISRJ leads to uneven the amplitude distribution of multiple false targets, which has both deception jamming and blanket jamming [34]. However, the interference effect has not been significantly improved. Furthermore, the research on combined jamming modulation methods for phase-coded signals is limited.

In order to overcome the drawbacks of ISRJ, forming controllable pre-lead false targets and blanket jamming for both LFM and phase-coded signals, an ISRJ based on joint frequency shift/phase modulation is proposed. Its frequency shift and two-phase modulation are controlled by the code sequence. The appropriate frequency shift matrix and phase modulation parameters can be selected for joint frequency shift/phase modulation to simultaneously generate a pre-lead false target and blanket jamming for LFM and phase-coded signals. The interference effect advantages for different signals are different.

- For LFM signals, joint modulation solves the problem of the same time–frequency distribution within an interrupted sampling period and destroys the distribution law of the pulse compression result. The coherent superposition of jamming signals is achieved by controlling the frequency shift matrix and phase modulation parameters. The strong false target or interference area is formed before the real target.
- For the phase-coded signals, the frequency shift modulation is reduced, and the pre-lead false target is generated using code sequence prediction and two-phase modulation. In addition, the sidelobe is similar to the noise interference, and the interference range is increased.

The rest of this paper is organized as follows. In Section 2, the principles of the ISRJ and the matched filtering characteristics of the ISRJ for LFM and phase-coded signals are introduced. Section 3 establishes the ISRJ model based on frequency shift and phase modulation, and analyzes the influence of modulation parameters on the interference effect. Section 4 analyzes the interference effect of ISRJ based on joint frequency shift/phase modulation. The simulation analysis is carried out in Section 5, and the paper is concluded in Section 6.

## 2. The Principle of ISRJ

### 2.1. ISRJ

**Figure 1:**
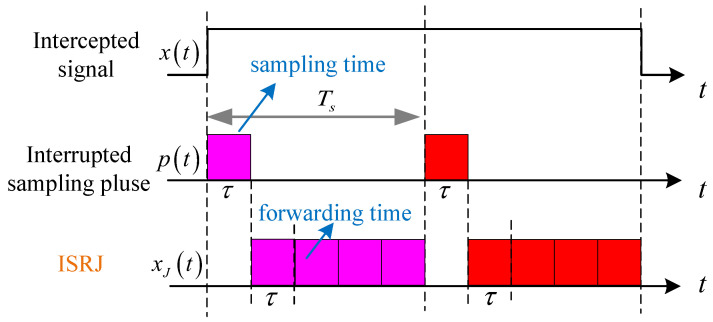
The principle of ISRJ.

The core idea of ISRJ is that the jammer intercepts the radar signal, periodically samples a small segment of the signal, and retransmits it in a period until the end of the pulse signal in Figure 1. ISRJ can be generated with a rectangular pulse train of equal width, and interrupted sampling pulse can be written as

(1) $$p\left(t\right)=\mathrm{rect}\left(t/\tau \right)*\sum_{n=-\infty }^{\infty }\delta \left(t-nT_{s}\right)$$

where ∗ is the convolution calculation, τ is the interrupted sampling pulse width, and $T_{s}\left(T_{s}\geq 2\tau \right)$ is the interrupted sampling period. The interrupted sampling frequency is $f_{s}=1/T_{s}$, and the duty cycle is $\lambda =\tau /T_{s}$. Subsequently, the forwarding times are $M_{J}=\left[1/\lambda -1\right]$, and the number of interrupted sampling periods is $N_{J}=\left\lfloor T_{p}/T_{s}\right\rfloor$. The Fourier series of the interrupted sampling pulse is provided as [23]

(2) $$\begin{array}{cc} p(t) & =\frac{\tau }{T_{s}}\cdot \sum_{n=1}^{\infty }Sa\left(\pi nf_{s}\tau \right)e^{j2\pi nf_{s}t}\\ \; & =\frac{\tau }{T_{s}}+2\frac{\tau }{T_{s}}\sum_{n=1}^{\infty }Sa\left(\pi nf_{s}\tau \right)cos2\pi nf_{s}t \end{array}$$

According to (1), using the Fourier transform rect(t/τ)↔τSa(πfτ), $\sum_{n=-\infty }^{\infty }\delta \left(t-nT_{s}\right)↔1/T_{s}\sum_{n=-\infty }^{\infty }\delta \left(f-nf_{s}\right)$, Sa(x)=sin(x)/x. The frequency spectrum of p(t) is provided as [3]

(3) $$P\left(f\right)=\sum_{n=-\infty }^{\infty }\tau \cdot Sa\left(\pi f\tau \right)\times \frac{1}{T_{s}}\cdot \delta \left(f-nf_{s}\right)=\sum_{n=-\infty }^{\infty }a_{n}\times \delta \left(f-nf_{s}\right)$$

where $a_{n}=\tau /T_{s}\cdot \mathrm{Sa}\left(\pi nf_{s}\tau \right)$. Assuming that the radar signal is x(t), ISRJ is expressed as follows:

(4) $$x_{J}\left(t\right)=x\left(t\right)p\left(t\right)$$

and its frequency spectrum is $X_{J}\left(f\right)=X\left(f\right)*P\left(f\right)$. Substituting (3) into (4), we arrive at the formula

(5) $$X_{J}\left(f\right)=\sum_{n=-\infty }^{\infty }a_{n}X\left(f-nf_{s}\right)$$

### 2.2. Analysis of ISRJ Parameters for LFM Signals

Considering the LFM signal as the intercepted radar signal, it can be expressed as

(6) $$x_{L}\left(t\right)=\mathrm{rect}\left(t/T_{p}\right)\times \exp\left[j2\pi f_{0}t+j\pi k_{f}t^{2}\right],t\leq \left|T_{p}/2\right|$$

where $f_{0}$ is the carrier frequency, $T_{p}$ is the pulse width, *B* is the bandwidth, and $k_{f}=B/T_{p}$ is the frequency modulation slope. The *m*-th forwarding delay time is $t_{d,m}=m\tau$, and the matched filter is $h\left(t\right)=x^{*}\left(-t\right)$. According to (2) and (4), the pulse compression result of ISRJ for the LFM signal is provided as

(7) $$\begin{array}{cc} y_{JL}\left(t\right) & =\sum_{m=1}^{M_{J}}x_{J}\left(t-t_{d,m}\right)*h\left(t\right)\\ \; & =\sum_{m=1}^{M_{J}}\sum_{n=1}^{\infty }\frac{\tau }{T_{s}}Sa\left(\pi nf_{s}\tau \right)e^{j2\pi nf_{s}\left(t-m\tau \right)}\cdot x_{L}\left(t-m\tau \right)*h\left(t\right)\\ \; & =\sum_{m=1}^{M_{J}}\left\{\frac{\tau }{T_{s}}+2\frac{\tau }{T_{s}}\sum_{n=1}^{\infty }Sa\left(\pi nf_{s}\tau \right)\cos\left[2\pi nf_{s}\left(t-m\tau \right)\right]\right\}\cdot x_{L}\left(t-m\tau \right)*h\left(t\right) \end{array}$$

Assuming $y_{m}\left(t\right)=x_{L}\left(t-m\tau \right)*h\left(t\right)$, the first part of (7) generates a zero-order false target, whose amplitude is directly proportional to the interrupted sampling pulse width and inversely proportional to the interrupted sampling period. The second part of (7) generates a sub-false target group. The frequency spectrum of $y_{m}\left(t\right)$ moves to each harmonic $nf_{s}$ of the rectangular pulse. Therefore, the sub-false target group formed by the *m*-th delay forwarding is symmetrically distributed around the zero-order false target. Its frequency spectrum is $Y_{JL}\left(f\right)=\sum_{m=1}^{M_{J}}X_{J}\left(f\right)e^{-j2\pi fm\tau }\cdot H\left(f\right)$. Since $H\left(f\right)=X_{L}^{*}\left(f\right)$, according to (5), we arrive at the formula

(8) $$Y_{JL}\left(f\right)=\sum_{n=-\infty }^{\infty }\sum_{m=1}^{M_{J}}a_{n}X_{L}\left(f-nf_{s}\right)X_{L}^{*}\left(f\right)e^{-j2\pi fm\tau }$$

Assuming $y_{n}\left(t\right)=F^{-1}\left[\sum_{m=1}^{M_{J}}X_{L}\left(f-nf_{s}\right)X_{L}^{*}\left(f\right)e^{-j2\pi fm\tau }\right]$, $F^{-1}$ means inverse FT. According to the definition of the ambiguity function, the amplitude of $y_{n}\left(t\right)$ is [5]

(9) $$\left|y_{n}\left(t\right)\right|=\sum_{m=1}^{M_{J}}\left|Sa\left\{\pi k_{f}\left(t-m\tau +\frac{nf_{s}}{k_{f}}\right)\left(T_{p}-\left|t-m\tau \right|\right)\right\}\cdot \left(1-\frac{\left|t-m\tau \right|}{T_{p}}\right)\right|$$

According to the characteristics of the Sa function, the peak output of $|y_{n}\left(t\right)|$ is $|y_{n}{\left(t\right)|}_{peak}=|1-|nf_{s}\left|/B\right|$ when $t=m\tau -nf_{s}/k_{f}$. Therefore, the time position of the *n*-order false target and the interval of the adjacent order false target by the *m*-th delay forwarding are expressed as

(10) $$\left\{\begin{array}{c} {\left(t_{m}\right)}_{n}=m\tau -nf_{s}/k_{f}\\ \Delta t_{m}=\left|f_{s}/k_{f}\right| \end{array}\right.$$

According to (7)–(9), when $t=m\tau -nf_{s}/k_{f}$, the output envelope of ISRJ is provided as

(11) $$\left|y_{JL}\left(t\right)\right|=\sum_{n=-\infty }^{\infty }\left|a_{n}\right|\cdot {\left|y_{n}\left(t\right)\right|}_{peak}$$

Therefore, the amplitude of the *n*-order false target is provided as

(12) $${\left(A_{JL}\right)}_{n}=\left|a_{n}\right|\cdot |1-|nf_{s}\left|/B\right|\cdot A_{x}\cdot \sqrt{10^{\left(JSR/10\right)}}$$

where ${\left(A_{JL}\right)}_{n}$ represents the amplitude of the *n*-order false target, and $A_{x}$ is the envelope of the real target. When n=0, the maximum value of ISRJ is $\tau /T_{s}$.

According to (10) and (12), the main factors influencing the position, amplitude, and interval of the false target are mainly the interrupted sampling pulse width and interrupted sampling period. The former is proportional to the amplitude of the false target. The larger it is, the farther the multi-order false target lags. The latter is inversely proportional to the false target amplitude and interval, affecting the position of the multi-order false target. When $f_{s}>0$, the position of the *n*-order false target is moved forward; otherwise, the position of the *n*-order false target is moved backward.

### 2.3. Analysis of ISRJ Parameters for Phase-Coded Signals

Considering that the intercepted radar signal is a two-phase coded signal, we obtain

(13) $$x_{c}\left(t\right)=\frac{1}{\sqrt{T_{c}}}\mathrm{rect}\left(\frac{t-T_{c}/2}{T_{c}}\right)*\frac{1}{\sqrt{P}}\sum_{w=0}^{P-1}C_{w}\cdot \delta \left(t-wT_{c}\right)$$

where $C_{w}=+1,-1$ is the binary code, $T_{c}$ is the code width, and *P* is the code length. Using the Fourier transform $\mathrm{rect}\left(\frac{t-T_{c}/2}{T_{c}}\right)↔Sa\left(\pi fT_{c}\right)exp\left(-j\pi fT_{c}\right)$, $\sum_{w=0}^{P-1}C_{w}\cdot \delta \left(t-wT_{c}\right)↔1/T_{c}\cdot \sum_{w=0}^{P-1}C_{w}exp\left(-j2\pi fwT_{c}\right)$, the frequency spectrum of $x_{c}\left(t\right)$ is provided as

(14) $$X_{c}\left(f\right)=\sqrt{T_{c}}\mathrm{Sa}\left(\pi fT_{c}\right)\exp\left(-j\pi fT_{c}\right)\cdot \frac{1}{\sqrt{P}}\sum_{w=0}^{P-1}C_{w}\exp\left(-j2\pi fwT_{c}\right)$$

The pulse compression results of $x_{c}\left(t\right)$ can be expressed as [29]

(15) $$\begin{array}{c} y_{c}\left(t\right)=\left\{\begin{array}{c} \left[1-\frac{\left(t-\left\lfloor t/T_{c}\right\rfloor \cdot T_{c}\right)}{T_{c}}\right]\cdot \frac{1}{P}\sum_{w=0}^{P-1-\left\lfloor t/T_{c}\right\rfloor }C_{w}C_{w+\left\lfloor t/T_{c}\right\rfloor },t\geq 0\\ \left[1-\frac{\left(t-\left\lceil t/T_{c}\right\rceil \cdot T_{c}\right)}{T_{c}}\right]\cdot \frac{1}{P}\sum_{w=-\left\lceil t/T_{c}\right\rceil }^{P-1}C_{w}C_{w+\left\lceil t/T_{c}\right\rceil },t<0 \end{array}\right. \end{array}$$

where $\left\lfloor \cdot \right\rfloor$ indicates rounding down, and $\left\lceil \cdot \right\rceil$ indicates rounding up. $\left[1-\frac{\left(t-\left\lfloor t/T_{c}\right\rfloor \cdot T_{c}\right)}{T_{c}}\right]$ represents a sawtooth wave with an amplitude less than one. $\frac{1}{P}\sum_{w=0}^{P-1-\lfloor t/T_{c}\rfloor }C_{w}\cdot C_{w+\lfloor t/T_{c}\rfloor }$ represents the normalized autocorrelation function of the code sequence, which is only related to the code.

Because the group delay characteristic of the waveform is opposite to the matched filter, the phase characteristic of the input signal is compensated by the matched filter. The peak output can be moved forward to the phase-coded signal if the jammer can change the group delay characteristics of the input signal through modulation and forwarding methods. The phase-coded signal group delay is provided as [30]

(16) $$\begin{array}{cc} t_{d}\left(f\right) & =\frac{1}{2\pi }\frac{d}{df}\arg\left(X_{c}\left(f\right)\right)\\ \; & =\frac{1}{2\pi }\frac{d}{df}\arg\left(\sqrt{T_{c}}\mathrm{Sa}\left(\pi fT_{c}\right)\exp\left(-j\pi fT_{c}\right)\cdot \frac{1}{\sqrt{P}}\sum_{w=0}^{P-1}C_{w}\exp\left(-j2\pi fwT_{c}\right)\right)\\ \; & =\frac{1}{2\pi }\frac{d}{df}\arg\left[\sum_{w=0}^{P-1}C_{w}\exp\left(-j2\pi f\left(w+1/2\right)T_{c}\right)\right] \end{array}$$

where the group delay characteristic of phase-coded signals is determined by the code and code width, and is independent of the frequency. Therefore, the frequency shift modulation cannot realize the false target forward.

Based on the relationship between the interrupted sampling pulse width and the code width, the sampling signal is divided into *P* sub-pulses with a code width of $T_{c}$. Therefore, the interrupted sampling pulse for phase-coded signal from (1) can be expressed as

(17) $$p_{c}\left(t\right)=\sum_{w=0}^{P-1}a_{w}\cdot \mathrm{rect}\left(\frac{t-T_{c}/2-wT_{c}}{T_{c}}\right)$$

where $a_{w}$ is the amplitude of the *w*-th sub-pulse, which is either 0 or 1. Therefore, in (17), the influence of the interrupted sampling pulse width and code width on $a_{w}$ is analyzed in the following three cases: (1) the sub-pulse is completely within the sampling time, so $a_{w}=1$; (2) the sub-pulse is completely in the forwarding time, $a_{w}=0$; and (3) if more than 50% of the sub-pulse is within the sampling time, $a_{w}=1$; otherwise $a_{w}=0$. Therefore, the pulse compression result of ISRJ for the phase-coded signal is expressed as follows [28]

(18) $$\begin{array}{c} y_{JC}\left(t\right)=\left\{\begin{array}{c} \left[1-\frac{\left(t-\left\lfloor t/T_{c}\right\rfloor \cdot T_{c}\right)}{T_{c}}\right]\cdot \frac{1}{P}\sum_{w=0}^{P-1-\left\lfloor t/T_{c}\right\rfloor }a_{w}C_{w}C_{w+\left\lfloor t/T_{c}\right\rfloor },t\geq 0\\ \left[1-\frac{\left(t-\left\lceil t/T_{c}\right\rceil \cdot T_{c}\right)}{T_{c}}\right]\cdot \frac{1}{P}\sum_{w=-\left\lceil t/T_{c}\right\rceil }^{P-1}a_{w}C_{w}C_{w+\left\lceil t/T_{c}\right\rceil },t<0 \end{array}\right. \end{array}$$

Compared with (15) and (18), the ISRJ for the phase-coded signal produces a lagging false target. The amplitude characteristics of the false target depend on the second half, which is equivalent to the weighted sum of the autocorrelation function of the code. The number of one in $a_{w}$ is determined by the duty cycle. Therefore, the larger the duty cycle, the higher the false target amplitude. The maximum value of the amplitude is $|y_{JC}{\left(t\right)|}_{max}=\tau f_{s}$. The false target lag time is determined by the interrupted sampling width. The smaller it is, the more timely the jamming signal is transmitted, and the shorter the false target lag time is.

## 3. Frequency Shift and Phase Modulation Characteristics of ISRJ

### 3.1. Frequency Shift and Phase Modulation Characteristics of ISRJ for LFM Signals

#### 3.1.1. Frequency Shift Modulatin Characteristics

The frequency shift modulation of ISRJ adopts the subsection frequency shift of the code sequence. Firstly, the code sequence in the interrupted sampling period is marked. For simplicity, $\left[iT_{s},\left(i+1\right)T_{s}\right]$ is regarded as the *i*-th interrupted sampling period, and the code sequence is from 0 to $n_{0}-1$ and is marked as $c_{i,0}$ to $c_{i,n_{0}-1}$. Therefore, the subsection frequency shift is provided as

(19) $$f_{d}\left(t\right)=\mathrm{rect}\left(\frac{t}{T_{f}}\right)*\left[\sum_{i=0}^{N_{J}-1}\sum_{k=0}^{n_{0}-1}c_{i,k}\times \xi_{i,k}\times \delta \left(t-iT_{s}-kT_{f}\right)\right]$$

where $c_{i,k}=1$ or 0. When $c_{i,k}=1$, it means there is frequency shift modulation; when $c_{i,k}=0$, there is no frequency shift modulation. $T_{f}$ is the frequency shift modulation code sequence width, and $\xi_{i,k}$ is the frequency shift controlled by the *k*-th code sequence in the *i*-th interrupted sampling period. Therefore, the frequency shift modulation function is expressed as

(20) $$u\left(t\right)=e^{j2\pi f_{d}\left(t\right)t}$$

**Figure 2:**
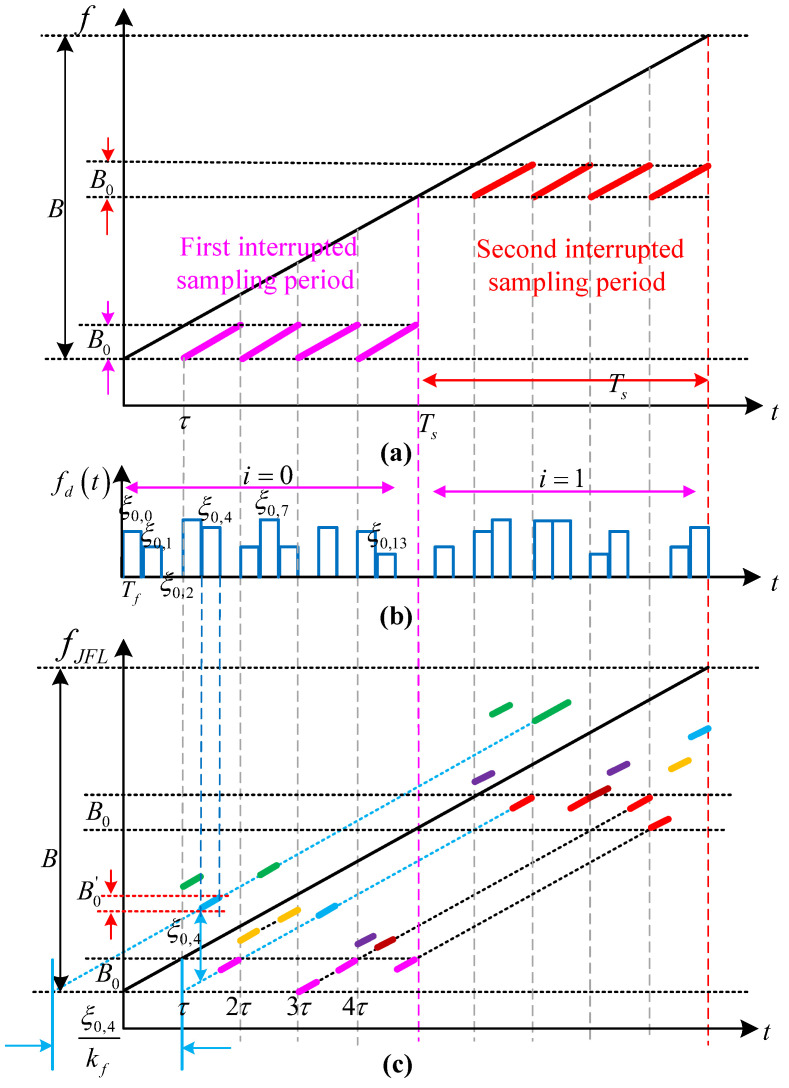
Timing diagram of ISRJ based on code-sequence-segmented frequency shift modulation: (**a**) the time–frequency distribution of ISRJ; (**b**) the time–domain distribution of the subsection frequency shift; and (**c**) the time–frequency distribution of ISRJ modulated by code-sequence-segmented frequency shift.

The ISRJ signal after frequency shift modulation is considered as having no mismatch loss. Assume the interference bandwidth of the ISRJ sub-pulse is $B_{0}=k_{f}\tau$, and the jamming bandwidth formed by the code sequence frequency shift modulation is $B_{0}^{{}^{\prime }}=k_{f}T_{f}$, as shown in Figure 2. The time–frequency distribution of ISRJ is discontinuous in Figure 2a, and the time–frequency distribution in each interrupted sampling period is the same. Figure 2b depicts the segmented frequency shift distribution controlled by the code sequence. The time–frequency distribution of ISRJ is modulated by code-sequence-segmented frequency shifts, which are distributed in front of, near, and behind the real target. Furthermore, multiple frequency-shifted slices can be accumulated at the same position to enhance the interference amplitude.

According to (10) and (12), the amplitude and position of the false target are, respectively, expressed as follows:

(21) $$t_{i,k}=\left\lfloor \frac{t-iT_{s}}{\tau }\right\rfloor \times \tau -\frac{\xi_{i,k}}{k_{f}}$$

(22) $${\left(A_{JFL}\right)}_{i,k}\approx B_{0}^{\prime }/B\cdot A_{x}\cdot \sqrt{10^{\left(JSR/10\right)}}=T_{f}/T_{p}\cdot A_{x}\cdot \sqrt{10^{\left(JSR/10\right)}}$$

where ${\left(A_{JFL}\right)}_{i,k}$ and $t_{i,k}$ represents the amplitude and position of the false target modulated by the *k*-th code sequence segmented frequency shift in the *i*-th interrupted sampling period, respectively. If $\xi_{i,k}>0$, the false target moves forward; otherwise, it moves backward. The positions of the false target generated by any two code-sequence-segmented frequency shift modulations are, respectively, provided as

(23) $$\left\{\begin{array}{c} t_{i_{0},k_{0}}=\left\lfloor \frac{t-i_{0}T_{s}}{\tau }\right\rfloor \times \tau -\frac{\xi_{i_{0},k_{0}}}{k_{f}}\\ t_{i_{{}^{\prime }},k_{{}^{\prime }}}=\left\lfloor \frac{t-i_{{}^{\prime }}T_{s}}{\tau }\right\rfloor \times \tau -\frac{\xi_{i_{{}^{\prime }},k_{{}^{\prime }}}}{k_{f}} \end{array}\right.$$

When $t_{i_{0},k_{0}}=t_{i_{{}^{\prime }},k_{{}^{\prime }}}$, the two false targets are coherently superimposed at the same position to achieve higher amplitude false targets. Assume $\left\lfloor \left(t-i_{0}T_{s}\right)/\tau \right\rfloor =\alpha_{0}$, $\left\lfloor \left(t-i_{{}^{\prime }}T_{s}\right)/\tau \right\rfloor =\alpha_{{}^{\prime }}$, and $\alpha_{0},\alpha_{{}^{\prime }}\left(0\leq \alpha_{0}\leq M_{J},0\leq \alpha_{{}^{\prime }}\leq M_{J},\alpha_{0}\leq \alpha_{{}^{\prime }}\right)$ are integers. If $t_{i_{0},k_{0}}=t_{i_{{}^{\prime }},k_{{}^{\prime }}}$, $\alpha_{0}=\alpha_{{}^{\prime }}$, then $\xi_{i_{0},k_{0}}-\xi_{i_{{}^{\prime }},k_{{}^{\prime }}}=0$. If $t_{i_{0},k_{0}}=t_{i_{{}^{\prime }},k_{{}^{\prime }}}$, $\alpha_{{}^{\prime }}=\alpha_{0}+\alpha_{1}$ ($\alpha_{1}<M_{J}$ and is an integer), then $\xi_{i_{0},k_{0}}-\xi_{i_{{}^{\prime }},k_{{}^{\prime }}}=\alpha_{1}k_{f}\tau$. Therefore, the relationship between any two code-sequence-segmented frequency shifts satisfies

(24) $$\xi_{i_{0},k_{0}}-\xi_{i_{{}^{\prime }},k_{{}^{\prime }}}=\left(\alpha_{{}^{\prime }}-\alpha_{0}\right)\times k_{f}\tau$$

The maximum code sequence segmented frequency shift is ${\left[\xi_{i,k}\right]}_{max}=M_{J}k_{f}\tau$ to ensure that the frequency shift modulation has no mismatch loss. Assuming the code sequence segmented frequency shift matrix is $\xi =[\xi_{0,0},\xi_{0,1},\cdots ,\xi_{0,n_{0}-1},\xi_{1,0},\cdots ,\xi_{N_{J}-1,n_{0}-1}]$, there are $\kappa_{1}$ code sequence frequency shift modulations coherently superimposed at the same position. According to (22), the maximum amplitude of the accumulated interference signal is provided as

(25) $${\left(A_{JFL}\right)}_{\kappa_{1},max}=\kappa_{1}\cdot \left(T_{f}/T_{P}\right)\cdot A_{x}\cdot \sqrt{10^{\left(JSR/10\right)}}$$

#### 3.1.2. Phase Modulation Characteristics

The ISRJ adopts the two-phase code sequence modulation. The phase modulation function is provided as

(26) $$\varphi \left(t\right)=\mathrm{rect}\left(\frac{t}{T_{\varphi }}\right)*\left[\sum_{l=0}^{P_{\varphi }-1}b_{l}\cdot \delta \left(t-lT_{\varphi }\right)\right]$$

where $b_{l}$ is the additional phase modulated by the *l*-th code sequence, $b_{l}=-1,1,0$. $T_{\varphi }$ is the phase modulation code sequence width, and $P_{\varphi }$ is the phase modulation code length. Generally, the width of the phase modulation code sequence should meet $T_{\varphi }<\tau$ to ensure that the interference signal phase jumps. Therefore, it can be ensured that the intercepted radar signal is controlled by more than two additional phases of the code sequence within an interrupted sampling pulse width. When the phase modulated by two adjacent code sequences is the same, the phase of the interference signal is consistent; otherwise, there is a maximum phase jump π. Assume $\tau =\chi T_{\varphi }$ (χ≥2 and is an integer), then the interrupted sampling pulse of ISRJ based on two-phase modulation from (1) is as follows.

(27) $$\begin{array}{cc} p_{JP}\left(t\right) & =\sum_{l_{1}=0}^{\chi -1}\mathrm{rect}\left(\frac{t-l_{1}T_{\varphi }}{T_{\varphi }}\right)*\sum_{l=0}^{P_{\varphi }-1}b_{l}\cdot \delta \left(t-m\left(l\right)T_{s}-\left\lfloor \frac{lT_{\varphi }-m\left(l\right)T_{s}}{\chi T_{\varphi }}\right\rfloor \chi T_{\varphi }\right) \end{array}$$

where $l_{1}$ represents the multiple relationship between τ and $T_{\varphi }$, $m\left(l\right)=\left\lfloor lT_{\varphi }/T_{s}\right\rfloor$. Using the Fourier transform $\delta \left(t-m\left(l\right)T_{s}-\left\lfloor \frac{lT_{\varphi }-m\left(l\right)T_{s}}{\chi T_{\varphi }}\right\rfloor \chi T_{\varphi }\right)↔exp\left\{-j2\pi f\left(m\left(l\right)T_{s}+\left\lfloor \frac{lT_{\varphi }-m\left(l\right)T_{s}}{\chi T_{\varphi }}\right\rfloor \chi T_{\varphi }\right)\right\}$, $\mathrm{rect}\left(\frac{t-l_{1}T_{\varphi }}{T_{\varphi }}\right)↔T_{\varphi }Sa\left(\pi fT_{\varphi }\right)exp\left(-j2\pi fl_{1}T_{\varphi }\right)$, the frequency spectrum is provided as

(28) $$\begin{array}{cc} P_{JP}\left(f\right) & =T_{\varphi }Sa\left(\pi fT_{\varphi }\right)\times \sum_{l_{1}=0}^{\chi -1}\sum_{l=0}^{P_{\varphi }-1}b_{l}\cdot exp\left\{-j2\pi f\left(l_{1}T_{\varphi }+m\left(l\right)T_{s}+\left\lfloor \frac{lT_{\varphi }-m\left(l\right)T_{s}}{\chi T_{\varphi }}\right\rfloor \chi T_{\varphi }\right)\right\}\\ \; & =T_{\varphi }Sa\left(\pi fT_{\varphi }\right)\times \beta \left(f\right) \end{array}$$

where, $\beta \left(f\right)=\sum_{l_{1}=0}^{\chi -1}\sum_{l=0}^{P_{\varphi }-1}b_{l}\cdot exp\left\{-j2\pi f\left(l_{1}T_{\varphi }+m\left(l\right)T_{s}+\left\lfloor \frac{lT_{\varphi }-m\left(l\right)T_{s}}{\chi T_{\varphi }}\right\rfloor \chi T_{\varphi }\right)\right\}$. Due to $p_{JP}\left(t\right)$ being a finite signal on $\left[-T_{P}/2,T_{P}/2\right]$ in the time domain period of the intercepted signal, it is expanded by the Fourier series. It can be expressed as the weighted sum of countless sine and cosine functions, and its frequency spectrum can be written as [25]

(29) $$P_{JP}\left(f\right)=\sum_{n=-\infty }^{\infty }\left\{T_{\varphi }Sa\left(\pi nf_{p}T_{\varphi }\right)\times \beta \left(nf_{p}\right)\right\}\times \delta \left(f-nf_{p}\right)$$

where $f_{p}=1/T_{p}$. The frequency spectrum of ISRJ modulated by the two-phase for LFM is as follows:

(30) $$\begin{array}{cc} Y_{JPL}\left(f\right) & =F[p_{JP}\left(t\right)x_{L}\left(t\right)*h\left(t\right)]\\ \; & =P_{JP}\left(f\right)*X_{L}\left(f\right)\times H\left(f\right)\\ \; & =\sum_{n=-S}^{S}\left\{T_{\varphi }Sa\left(\pi nf_{p}T_{\varphi }\right)\times \beta \left(nf_{p}\right)\right\}\cdot X_{L}\left(f-nf_{p}\right)X_{L}^{*}\left(f\right) \end{array}$$

where h(t)↔H(f), $S=\left(B-f_{p}\right)/2f_{p}$, *F* means FT. Through the inverse Fourier transform, the envelope of the pulse compression result is provided as from (8) and (9).

(31) $$\left|y_{JPL}\left(t\right)\right|=\sum_{n=-S}^{S}\left|T_{\varphi }Sa\left(\pi nf_{p}T_{\varphi }\right)\beta \left(nf_{p}\right)\right|\times \left|1-\frac{\left|nf_{p}\right|}{B}\right|\times \left|Sa\left(\pi \left(t+\frac{nf_{p}}{k_{f}}\right)\left(B-\left|n\right|f_{p}\right)\right)\right|$$

According to (31), it can be observed that the envelope of the interference output is the amplitude and the phase-weighted superposition of 2S+1 Sinc functions, and the jamming effect depends on $|T_{\varphi }Sa\left(\pi nf_{p}T_{\varphi }\right)\cdot \beta \left(nf_{p}\right)|$. The interval of false targets depends on the spectral line interval of $|\beta (nf_{p})|$, and their distribution is consistent with the distribution of $|\beta (nf_{p})|$ on the frequency axis. According to (27), the adjustable parameters of ISRJ based on phase modulation include the interrupted sampling period, additional phase, phase modulation code sequence width, and χ. Assuming that the interrupted sampling period and χ are fixed, the jamming effect of the additional phase and the phase modulation code sequence width is analyzed in the following:

(1) The phase modulation code sequence width $T_{\varphi }$ mainly affects $\left|\beta (nf_{p})\right|$, $|T_{\varphi }Sa\left(\pi nf_{p}T_{\varphi }\right)|$, the effective false target amplitude, and interference range. However, the influence of $|T_{\varphi }Sa\left(\pi nf_{p}T_{\varphi }\right)|$ on the false target amplitude and the interference range are mutually restricted. Therefore, to increase the phase modulation effect, the influence of $|T_{\varphi }Sa\left(\pi nf_{p}T_{\varphi }\right)|$ on the interference amplitude should be reduced as much as possible. The weight of $\left|\beta (nf_{p})\right|$ should be increased in the amplitude adjustment.
(2) The additional phase $b_{l}$ affects $\left|\beta (nf_{p})\right|$, which works through the relationship between the additional phases, and the effect is complex.

### 3.2. Frequency Shift and Phase Modulation Characteristics of ISRJ for Phase-Coded Signals

#### 3.2.1. Frequency Shift Modulation Characteristics

According to (13), (17), and (20), ISRJ modulated by the code-sequence-segmented frequency shift for the phase-coded signal is expressed as

(32) $$\begin{array}{cc} x_{JFC}\left(t\right) & =x_{c}\left(t\right)\cdot p_{c}\left(t\right)\cdot u\left(t\right)\\ \; & =\sum_{w=0}^{P-1}\left\{\frac{1}{\sqrt{PT_{c}}}C_{w}\mathrm{rect}\left(\frac{t-T_{c}/2-wT_{c}}{T_{c}}\right)\right\}\times \left\{a_{w}\mathrm{rect}\left(\frac{t-T_{c}/2-wT_{c}}{T_{c}}\right)\right\}\times e^{j2\pi f_{d}\left(t\right)t}\\ \; & =\left\{\frac{1}{\sqrt{PT_{c}}}\sum_{w=0}^{P-1}a_{w}C_{w}\mathrm{rect}\left(\frac{t-T_{c}/2-wT_{c}}{T_{c}}\right)\right\}\times e^{j2\pi f_{d}\left(t\right)t} \end{array}$$

Because the ambiguity function of the pseudo-random code is similar to the pushpin-shaped code, the approximation degree increases with the increase in the time–bandwidth product. Therefore, for the phase-coded signal, the segmented frequency shift modulation causes a mismatch between the jamming signal and matched filter, and there is no pulse pressure result.

Considering the influence of the segmented frequency shift modulation mismatch, and assuming that the ISRJ signal with a ratio ϵ(0≤ϵ≤1) is not modulated by the segmented frequency shift. The maximum value of the interference output is expressed as follows based on (18):

(33) $${\left[y_{JFC}\left(t\right)\right]}_{max}={\left[y_{JC}\left(t\right)\right]}_{max}\cdot \epsilon =\tau f_{s}\cdot \epsilon$$

#### 3.2.2. Phase Modulation Characteristics

It is known from (17) and (26) that ISRJ modulated by two-phase code sequence for the phase-coded signal is provided as

(34) $$\begin{array}{cc} x_{JPC}\left(t\right) & =x_{c}\left(t\right)\cdot p_{c}\left(t\right)\cdot \varphi \left(t\right)\\ \; & =\left\{\frac{1}{\sqrt{PT_{c}}}\sum_{w=0}^{P-1}a_{w}C_{w}\cdot \mathrm{rect}\left(\frac{t-T_{c}/2-wT_{c}}{T_{c}}\right)\right\}\times \left\{\sum_{l=0}^{P_{\varphi }-1}b_{l}\cdot \mathrm{rect}\left(\frac{t-T_{\varphi }/2-lT_{\varphi }}{T_{\varphi }}\right)\right\} \end{array}$$

Assuming $T_{\varphi }=T_{c}$, there is $P_{\varphi }=P$. Therefore, the interference signal is provided as

(35) $$x_{JPC}\left(t\right)=\left\{\frac{1}{\sqrt{T_{c}}}\mathrm{rect}\left(\frac{t-T_{\varphi }/2}{T_{\varphi }}\right)\right\}*\left\{\frac{1}{\sqrt{P}}\sum_{w=0}^{P-1}a_{w}b_{w}C_{w}\cdot \delta \left(t-wT_{\varphi }\right)\right\}$$

From (13), (15), (18), and (35), the pulse compression result of $x_{JPC}\left(t\right)$ can be written as

(36) $$y_{JPC}\left(t\right)=\left\{\begin{array}{c} \left[1-\frac{\left(t-\left\lfloor t/T_{\varphi }\right\rfloor \cdot T_{\varphi }\right)}{T_{\varphi }}\right]\cdot \frac{1}{P}\sum_{w=0}^{P-1-\left\lfloor t/T_{\varphi }\right\rfloor }a_{w}b_{w}C_{w}C_{w+\left\lfloor t/T_{\varphi }\right\rfloor },t\geq 0\\ \left[1-\frac{\left(t-\left\lceil t/T_{\varphi }\right\rceil \cdot T_{\varphi }\right)}{T_{\varphi }}\right]\cdot \frac{1}{P}\sum_{w=-\left\lceil t/T_{\varphi }\right\rceil }^{P-1}a_{w}b_{w}C_{w}C_{w+\left\lceil t/T_{\varphi }\right\rceil },t<0 \end{array}\right.$$

Compared with (15), (18), and (36), since $a_{w}=1,0$, the pre-lead false target is realized by adjusting the phase modulation parameters $b_{w}$. Assuming that $C_{w+\left\lceil t/T_{\varphi }\right\rceil }=C_{w+\gamma }=a_{w}b_{w}C_{w}$ is satisfied after phase modulation, it is necessary to predict the following γ-th code in advance, and perform the phase modulation to produce the pre-lead false target. When the interrupted sampling pulse width is greater than the minimum sampling code sequence required to accurately predict the code, the code can be accurately predicted. Longer code sequences have relatively low sidelobes. However, the longer the code sequence for the jammer, the smaller the ratio between the minimum number of codes required for intermittent sampling and the total length of the codes. Furthermore, the longer the interference analysis time of the code sequence, the more accurate the code prediction. Therefore, when γ>0, the codes that have not been received are predicted and forwarded in advance to form a pre-lead false target. Suppose $\gamma_{m,i}$ is the code sequence interval that has not yet been received after the prediction in the *i*-th interrupted sampling period of the *m*-th forwarding. The predicted code sequence interval matrix is $\gamma =[\gamma_{0,0},\gamma_{0,1},\cdots ,\gamma_{0,N_{J}-1},\gamma_{1,0},\cdots ,\gamma_{M_{J},N_{J}-1}]$. The position of the pre-lead false target in the *i*-th interrupted sampling period of the *m*-th forwarding is expressed as

(37) $$t_{m,i}^{{}^{\prime }}=m\tau -\gamma_{m,i}\cdot T_{\varphi }$$

Assuming that $\kappa_{2}$ code sequences can be accurately and continuously predicted and forwarded, the maximum amplitude of the accumulated pre-lead false target is provided as follows from (18) and (36)

(38) $${\left(A_{JPC}\right)}_{\kappa_{2},max}=\frac{\kappa_{2}}{P}\cdot \tau f_{s}\cdot A_{x}\cdot \sqrt{10^{\left(JSR/10\right)}}$$

## 4. ISRJ Based on Joint Frequency Shift/Phase Modulation

For LFM, the phase modulation controls the amplitude and interval of the false target. With the increase in the phase modulation code sequence width, the amplitude and interval of false targets increase, and the number of effective false targets and interference range decrease, resulting in deception interference. On the contrary, blanket interference is formed. The pulse compression result of the code-sequence-segmented frequency shift modulation can form a strong pre-lead false target without any mismatch loss. In the case of the maximum forwarding times, the position with the largest forward distance and the maximum interference amplitude can be written as follows based on (21) and (25).

(39) $$\left\{\begin{array}{c} {\left[t_{i,k}\right]}_{min}=\left\lfloor \frac{t-iT_{s}}{\tau }\right\rfloor \times \tau -\frac{{\left[\xi_{i,k}\right]}_{max}}{k_{f}}=\left\lfloor \frac{t-iT_{s}}{\tau }\right\rfloor \times \tau -M_{J}\tau \\ {\left(A_{JFL}\right)}_{\max}=M_{J}\cdot \tau f_{s}\cdot A_{x}\cdot \sqrt{10^{\left(JSR/10\right)}} \end{array}\right.$$

For phase-coded signals, the code-sequence-segmented frequency shift modulation will cause matched filtering mismatch. According to the initial code sequence of the predicted codes and the number of continuously and accurately predicted codes, the phase modulation of the binary code sequence forms a pre-lead false target with a controllable position and amplitude.

**Figure 3:**
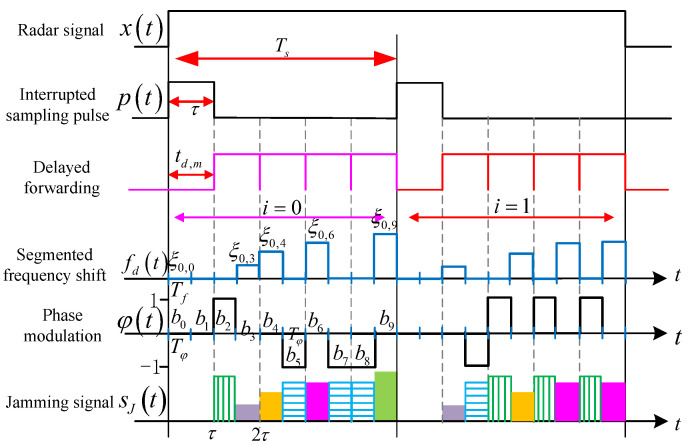
Principle block diagram of ISRJ based on joint frequency shift/phase modulation.

Therefore, an ISRJ controlled by code-sequence-segmented frequency shift and two-phase code sequence joint modulation is proposed. This method controls the position, amplitude, and density distribution of false targets by flexibly adjusting the frequency shift/phase modulation parameters. It can form a pre-lead false target for both LFM and phase-coded signals, destroy the ISRJ distribution law, and create deception and blanket interference, as shown in Figure 3.

The ISRJ based on joint frequency shift/phase modulation is the product of the intercepted radar signal, interrupted sampling pulse, frequency shift modulation function, and phase modulation function. This is written as

(40) $$s_{J}\left(t\right)=x\left(t\right)\times p\left(t\right)\times u\left(t\right)\times \varphi (t)$$

It is assumed that the code sequence segmental frequency shift modulation width and the code sequence phase modulation width are in a multiple relationship, which is that $T_{f}=\mu T_{\varphi }$ (μ is an integer). The interference performance of joint frequency shift and phase modulation is evaluated by analyzing whether there is a phase jump in the code sequence segmented frequency shift slice.

### 4.1. No Phase Jump in the Code Sequence Segmented Frequency Shift Slice

Ensure that there is no phase jump in the code-sequence-segmented frequency shift slice $c_{i,k}$ and then satisfy

(41) $$\left\{\begin{array}{c} c_{i,k}=1\\ b_{i\cdot n_{0}+k\cdot u}=b_{i\cdot n_{0}+k\cdot u+1}=\cdots =b_{i\cdot n_{0}+ku+u-1} \end{array}\right.$$

Therefore, the characteristics of the joint modulation are the same as those of only the frequency shift modulation. Assuming $\tau =\chi T_{f}=\chi T_{\varphi }$, ISRJ only has a proportion of η to participate in the frequency shift modulation, and 1−η of ISRJ participates in phase modulation. It can be noted from (39) that the maximum distance of the pre-lead false target is unchanged, and the maximum interference amplitude is expressed as

(42) $${\left(A_{JPFL}\right)}_{max}=\eta \times M_{J}\cdot \tau f_{s}\cdot A_{x}\cdot \sqrt{10^{\left(JSR/10\right)}}$$

where ${\left(A_{JPFL}\right)}_{max}$ represents the maximum amplitude of ISRJ based on joint frequency shift/phase modulation for the LFM signal. This part of the signal participating in frequency shift modulation cannot form the pulse compression result for phase-coded signals.

It is assumed that the ISRJ signal with a ratio 1−η participates in phase modulation. This part of the signal forms a delayed blanket jamming for the LFM signal. For phase-coded signals, this part continuously predicts multiple code sequences and forwards them in advance to generate the pre-lead false target. It can be seen from (37) and (38) that, when the codes involved in the phase modulation are all continuous and can be predicted accurately, the position with the largest forward distance and the maximum amplitude of the pre-lead false target are, respectively, expressed as

(43) $$\left\{\begin{array}{c} {\left[t_{m,i}^{{}^{\prime }}\right]}_{min}=m\tau -{\left[\gamma_{m,i}\right]}_{max}\cdot T_{\varphi }=m\tau -P\cdot \left(1-\eta \right)\cdot T_{\varphi }\\ {\left(A_{JPFC}\right)}_{max}=\left(1-\eta \right)\times \tau f_{s}\cdot A_{x}\sqrt{10^{\left(JSR/10\right)}} \end{array}\right.$$

where, ${\left(A_{JPFC}\right)}_{max}$ represents the maximum amplitude of ISRJ based on frequency shift/phase modulation for the phase-coded signal.

Therefore, in order to ensure the validity of the pre-lead false target, ${\left[t_{i,k}\right]}_{min}<0$, ${\left[t_{m,i}^{{}^{\prime }}\right]}_{max}<0$, ${\left(A_{JPFC}\right)}_{max}\geq A_{x}$, and ${\left(A_{JPFL}\right)}_{max}\geq A_{x}$. According to (39), (42), and (43), η is provided as

(44) $$\frac{1}{M_{J}\tau f_{s}\sqrt{10^{\left(JSR/10\right)}}}\leq \eta \leq 1-\frac{1}{\tau f_{s}\sqrt{10^{\left(JSR/10\right)}}}$$

### 4.2. Phase Jump in the Code Sequence Segmented Frequency Shift Slice

If there is a phase jump in the code-sequence-segmented frequency shift slice for the LFM signal, the corresponding phase change in the segmented frequency shift modulation will broaden the interference range of the pre-lead interference. It will also reduce the interference amplitude, but the distribution of the lagging false target remains unchanged.

For phase-coded signals, the phase change in the code-sequence-segmented frequency shift slice lead to a sharp decrease in the interference amplitude. Even though the 1−η of the ISRJ participates in the phase modulation and can accurately predict the code sequence, the interference performance caused by the frequency shift modulation is still not significantly improved.

**Figure 4:**
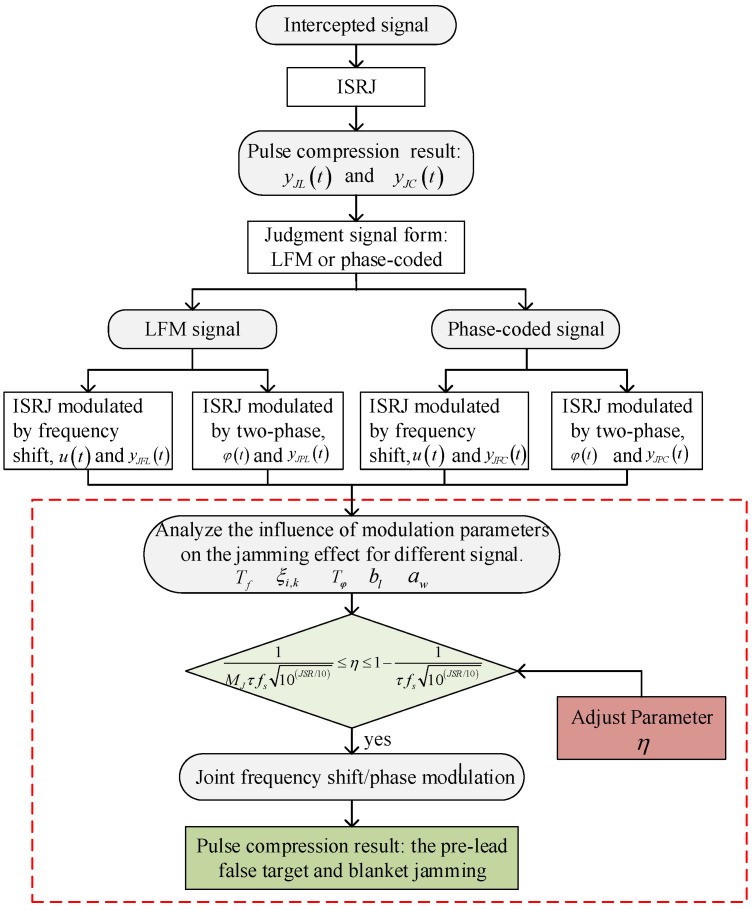
The workflow of ISRJ based on joint frequency shift/phase modulation.

Therefore, in order to adjust the modulation parameters as little as possible and have a good interference effect on both signals, it is generally ensured that there is no phase jump in the code-sequence-segmented frequency shift slice, and the frequency shift modulation proportion satisfies (44). Therefore, the workflow of the improved ISRJ in this paper is shown in Figure 4.

## 5. Simulation

The simulation results verify the advantages of this method, and the simulation parameters settings for ISRJ based on joint frequency shift/phase modulation are shown in Table 1.

**Figure 5:**
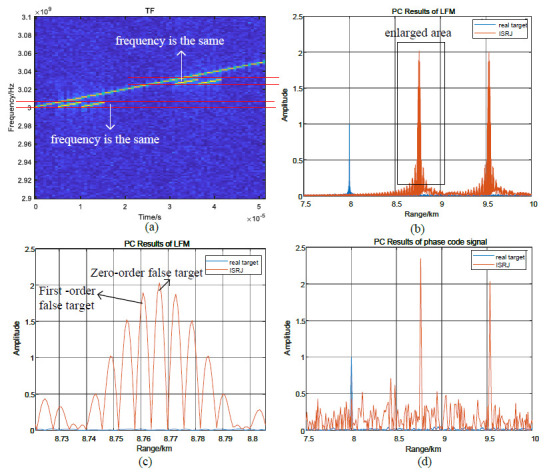
The distribution characteristics of ISRJ: (**a**) the time–frequency of ISRJ for LFM signal; (**b**) the pulse compression results of ISRJ for LFM signal; (**c**) the enlarged area in (**b**); and (**d**) the pulse compression results of ISRJ for phase-coded signal.

Figure 5 illustrates the time–frequency and the pulse compression result of ISRJ for the LFM signal and phase-coded signal. When $M_{J}=2$, the time–frequency distribution of the ISRJ for the LFM signal is discontinuous, and the time–frequency distribution is the same in each interrupted sampling period in Figure 5a. For the LFM signal, the two forwardings form two lagging false target strings, and the lagging distances are, respectively, τ and 2τ in Figure 5b. Zooming in on the region in Figure 5b, the false target string consists of a zero-order false target and multi-order false targets in Figure 5c. For the phase-coded signal, only two lagging false targets can be generated by two forwardings in Figure 5d.

### 5.1. Frequency Shift and Phase Modulation Characteristics of ISRJ

**Figure 6:**
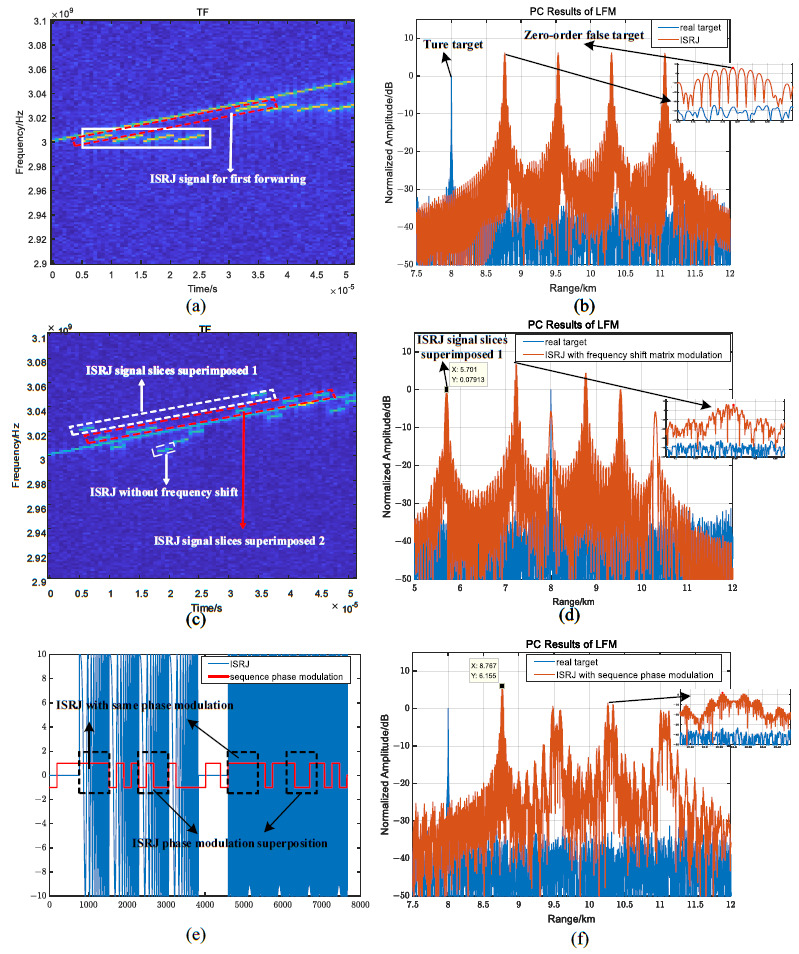
ISRJ, frequency shift, and phase modulation characteristics for LFM signals. (**a**) TF of ISRJ; (**b**) pulse compression result of ISRJ; (**c**) TF of ISRJ with frequency shift modulation; (**d**) pulse compression result of ISRJ with frequency shift modulation; (**e**) phase and waveform distribution of ISRJ with phase modulation; (**f**) and pulse compression result of ISRJ with phase modulation.

When $M_{J}=4$, Figure 6 illustrates the ISRJ, frequency shift modulation, and phase modulation characteristics of the LFM signal. It is obvious that the time–frequency distribution characteristics are the same in each interrupted sampling period in Figure 6a, and can be easily recognized using anti-interference methods with time–frequency analysis. Four groups of false target strings with the same distribution characteristics are generated through four delay forwardings, and the maximum amplitude of the zero-order false target is 6 dB, as shown in Figure 6b. For the frequency shift modulation characteristics, assuming $T_{f}=0.5\tau$, $n_{0}=10$, and the frequency shift matrix ξ=[552010515200101555102051515201015] MHz, the interference slice modulated by the segmented frequency shift is formed near the frequency modulation slope of the echo signal in Figure 6c. It is known from (25) that the coherent superposition of the code-sequence-segmented frequency shift interference slice on the same time–frequency distribution line can be achieved, which forms the pre-lead, nearby and lag the false target with a controllable position and amplitude. Because $\xi_{0,2}=\xi_{1,3}=20$ MHz, the two frequency shift interference slices are superimposed through the first forwarding in Figure 6c. The position and the maximum amplitude of the pre-lead false target are, respectively, $R_{0}+c\cdot \left(\tau -\xi_{0,2}/k_{f}\right)/2$ and 0 dB in Figure 6d. In the same way, the pre-lead false target amplitude formed by the coherent superposition of the six segmented frequency shift interference slices is 6.48dB. As shown in Figure 6e, when ISRJ has the same phase modulation in every forwarding, it does not affect the amplitude and position of the false target. On the contrary, when the phase jumps, the interval of the false target is changed, the amplitude of the zero-order false target is reduced, and the sidelobe amplitude is increased. Furthermore, the interference range is widened, and the regional blanket interference is formed.

**Figure 7:**
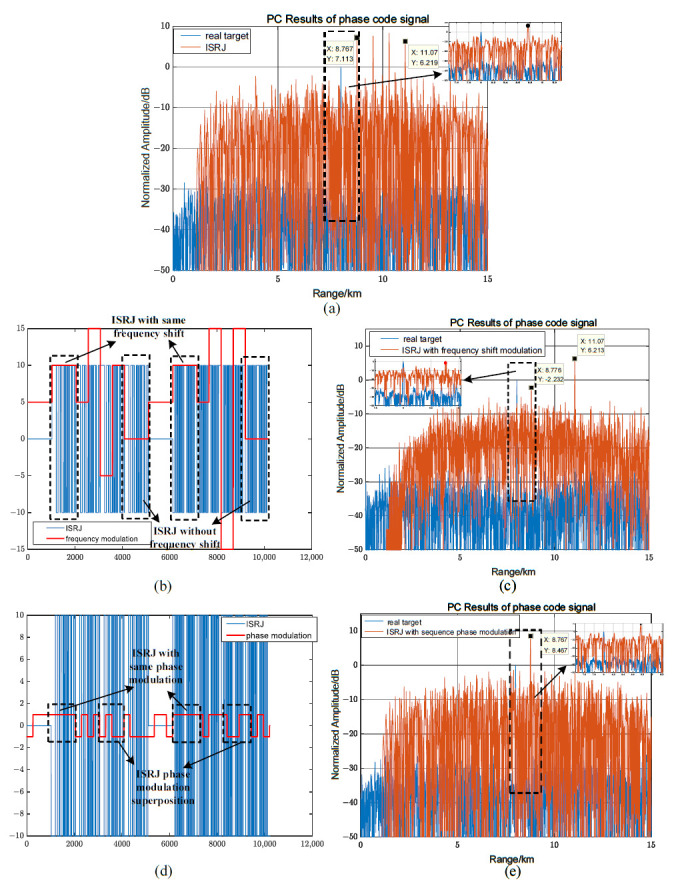
ISRJ, frequency shift, and phase modulation characteristics for phase-coded signals. (**a**) Pulse compression result of ISRJ; (**b**) Frequency shift distribution of ISRJ; (**c**) Pulse compression result of ISRJ with frequency shift modulation; (**d**) Phase and waveform distribution of ISRJ; and (**e**) Pulse compression result of ISRJ with phase modulation.

Assuming $M_{J}=4$, the phase-coded signal is a 511-bit M sequence with a code sequence width of $T_{c}=0.1\;$μs. Figure 7 illustrates the ISRJ, frequency shift modulation, and phase modulation characteristics for the phase-coded signals. Since the delay time of ISRJ for the *m*-th retransmission is mτ, the four high-fidelity false targets with fixed delay times are formed through four retransmissions, as shown in Figure 7a. The magnitude of the high-fidelity false target is 7 dB. For the frequency shift modulation characteristics, the segmented frequency shift of the code sequence within the pulse width of each delayed forwarding leads to a sharp drop in the false target amplitude in Figure 7b,c. Assume that the frequency shift matrix is ξ=[551010515−51000551010−515−151500] MHz. Since $\xi_{0,2}=\xi_{0,3}=\xi_{1,2}=\xi_{1,3}=10$ MHz, the frequency shift of the interference signal for the first delay forwarding is 10 MHz, resulting in the amplitude of the first high-fidelity false target dropping to −2.2 dB in Figure 7. Because $\xi_{0,8}=\xi_{0,9}=\xi_{1,8}=\xi_{1,9}=0$ MHz, the interference signal for the fourth forwarding has no frequency shift modulation, and the interference amplitude of the high-fidelity false target remains unchanged at 6.2 dB by comparing Figure 7a,c. As shown in Figure 7d,e, when the phase modulation does not jump for the first delay forwarding, there is no impact on the first delayed high-fidelity false target, and the amplitude increases to 8.5 dB. On the contrary, when the jump occurs, the amplitude of the lagging high-fidelity false target decreases sharply, and the sidelobe amplitude increases by about 2 dB.

**Figure 8:**
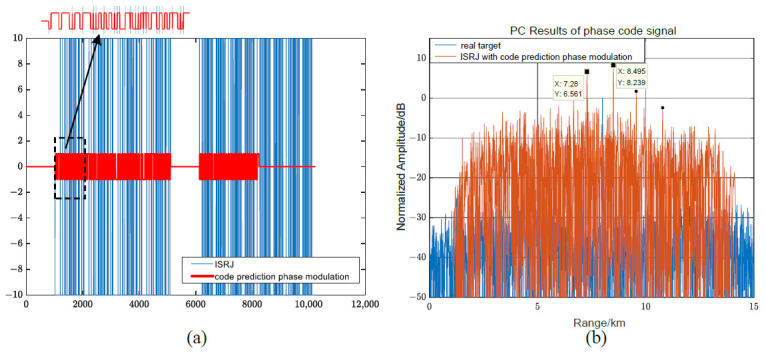
The code prediction phase modulation characteristics for phase-coded signals with γ=[001001007070500200]. (**a**) The code prediction phase distribution of ISRJ; and (**b**) pulse compression result of ISRJ based on the code prediction phase modulation.

Figure 8 depicts the influence of different code predictions on the pre-lead false target for the phase-coded signal. Suppose $T_{\varphi }=T_{c}=0.1$ μs, and the predicted code sequence interval matrix is γ=[001001007070500200]. Since the code sequence interval for first delayed forwarding is $\gamma_{1,0}=\gamma_{1,1}=100$, the pre-lead false target will be formed at $R_{0}+c\cdot \left(\tau -\gamma_{1,0}*T_{\varphi }\right)/2=$7.27 km with an amplitude of 6.5 dB according to (37). Since $\gamma_{4,0}=20$, $\gamma_{4,1}=0$, due to the small predicted code sequence interval, the amplitude of the pre-lead false target caused by the fourth delayed forwarding is relatively low in Figure 8b.

### 5.2. Frequency Shift/Phase Joint Modulation Characteristics of ISRJ

**Figure 9:**
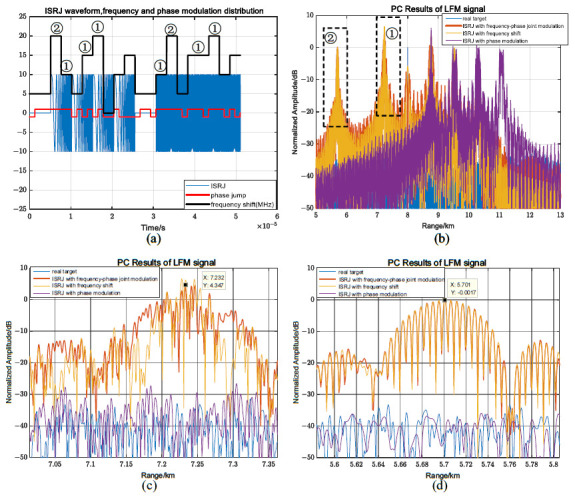
The frequency shift/phase joint modulation characteristics for LFM signals. (**a**) Jamming waveform, frequency shift, and phase modulation distribution of LFM signals; (**b**) PC results of interference; (**c**) enlargement area ➀; and (**d**) enlargement area ➁.

In Figure 9, the interference effects of the frequency shift and phase joint modulation on the LFM signal are introduced for $T_{f}=2T_{\varphi }$. The multiple-code-sequence-segmented frequency shift slices marked ➀ in Figure 9a form an accumulation at the region marked as ➀ in Figure 9b. Due to the phase jump of some of the code-sequence-segmented frequency shift slices, the jamming has the frequency shift modulation characteristics of generating the pre-lead false targets and the phase modulation characteristics of widening the jamming range, as shown in Figure 9c. The two segmented frequency shift interference slices marked as ➁ in Figure 9a have no phase jump, and the pre-lead false target has the same pulse compression result as the false target generated by only the frequency shift, as shown in Figure 9d.

**Figure 10:**
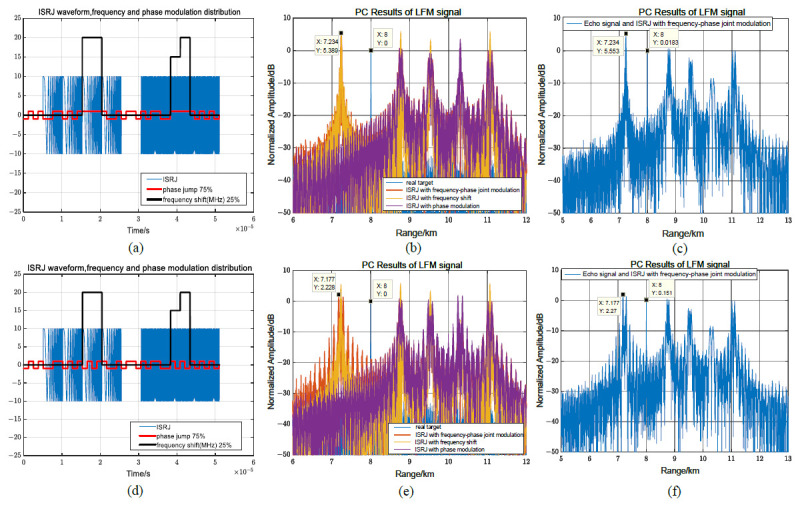
Influence of phase jumping on jamming performance in segmented frequency shift slices for LFM signals. (**a**–**c**) No phase jumps in segmented frequency shifting. (**d**–**f**) Phase jumps in segmented frequency shifting.

When $M_{J}$ = 4, according to (44), the proportion of the segmental frequency shift modulation is 0.125≤η≤0.5. For LFM signals, the proportions of the segmental frequency shift modulation and the phase jump in the segmental frequency shift have a greater impact on the interference performance. Assuming η = 0.25, $T_{f}=2T_{\varphi }$, there is no phase jump in the segmental frequency shift, and the maximum pre-lead false target amplitude is 5.4 dB. The maximum pre-lead false target amplitude generated by the received mixed signal remains unchanged, causing it to be difficult to identify the real target in Figure 10a–c. When there are phase jumps in the segmented frequency shift in Figure 10d, compared with Figure 10c, the position of the false target in Figure 10f remains unchanged, but the interference range of the pre-lead false target is widened due to the phase change, and the interference amplitude drops to 2.27 dB.

**Figure 11:**
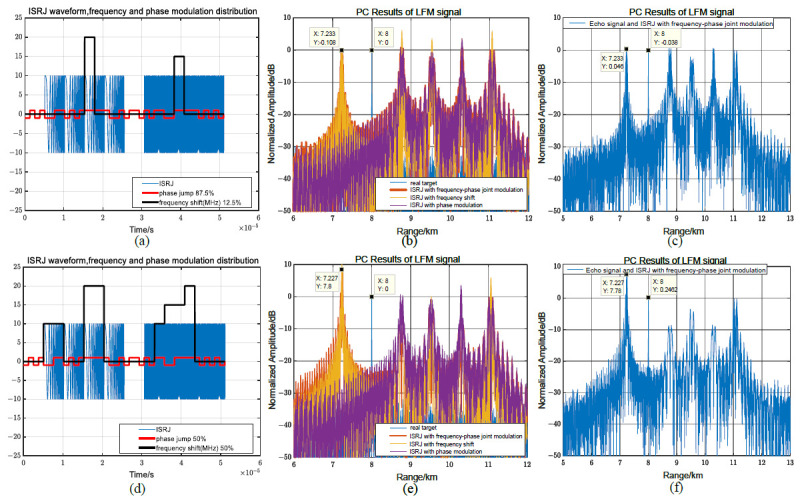
Influence of segmented frequency shift modulation ratio η on interference performance for LFM signals. (**a**–**c**) Segmented frequency shift modulation ratio η= 12.5%. (**d**–**f**) Segmented frequency shift modulation ratio η=50%.

Figure 11 illustrates the effect of the frequency shift modulation ratio on interference performance. When $M_{J}$ = 4, the proportion of the segmental frequency shift modulation is 0.125≤η≤0.5. Assuming η = 12.5%, the maximum amplitude of the pre-lead false target without a phase change in the frequency shift modulation is 0 dB, which is the same as the amplitude of the real target in Figure 11a–c. When η = 50%, the maximum magnitude of the pre-lead false target is 7.8 dB. Therefore, comparing Figure 10c and Figure 11c,f, as the proportion of the frequency shift modulation increases in the interval [0.125, 0.5], the magnitude of the pre-lead false target gradually increases.

**Figure 12:**
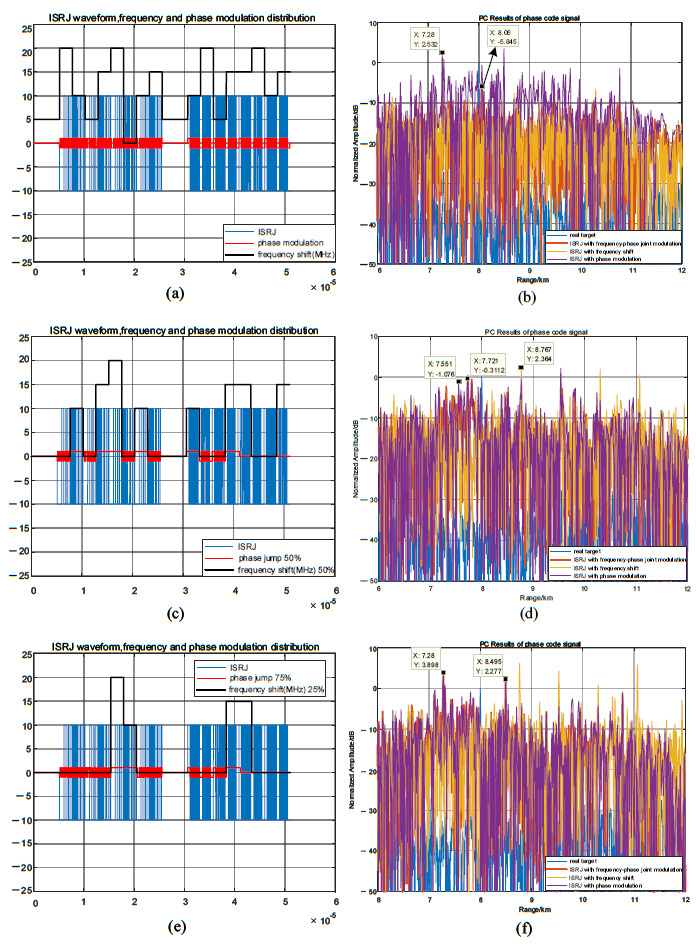
The joint frequency shift/phase modulation characteristics for phase-coded signals. (**a**,**c**,**e**) Jamming waveform, frequency shift, and phase modulation distribution. (**b**,**d**,**f**) PC results of interference.

Suppose $M_{J}$ = 4, 0.125≤η≤0.5, and $T_{\varphi }=T_{c}=0.1\;$μs, for phase-coded signals, there are frequency shifts and phase jumps in each delay forwarding, and the interference effect of the joint modulation is the same as that of only the frequency modulation, as shown in Figure 12a,b. Assuming that there is no phase jump in the interference signal modulated by the code sequence segmented frequency shift slice, the interference effect of the joint modulation is approximately equal to the interference effect of the phase modulation, as shown in Figure 12c,d. This is because the proportion of the phase modulation for code prediction is relatively small, the sidelobe signal weakens the amplitude of the pre-lead false target, and the position of the pre-lead false target changes slightly. In Figure 12e,f, the sidelobe is improved by increasing the proportion of phase modulation involved in the code prediction, and the amplitude of pre-lead false target is increased to about 4 dB using joint modulation. Compared with Figure 8, the interference effect is closer to the code prediction phase modulation interference, and the maximum amplitude is approximately 75% of the code prediction phase modulation interference.

### 5.3. Comparative Analysis of the Modulation Methods in This Paper and Other Modulation Methods

**Figure 13:**
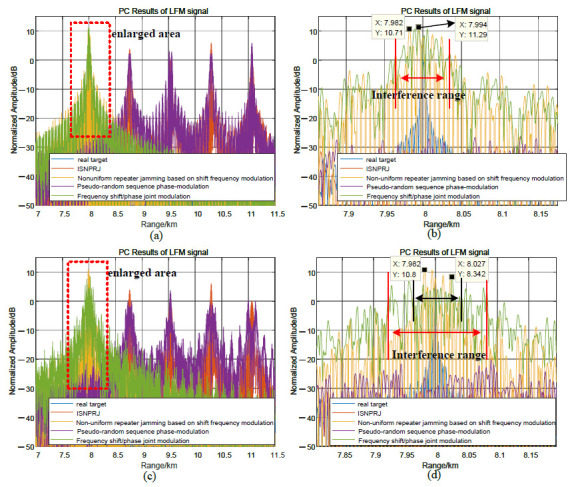
Comparative analysis of joint modulation methods and other methods for LFM signals. (**a**,**b**) When $T_{\varphi }=0.5T_{f}=2.555\;$μs, the pulse compression result of several modulation methods. (**c**,**d**) When $T_{\varphi }=0.1\;$μs, the pulse compression result of several modulation methods.

Figure 13 compares and analyzes the interference performance of the joint modulation algorithm and other modulation algorithms for LFM signals. When $T_{f}=5.11\;$μs, $T_{\varphi }=2.555\;$μs, $M_{J}=4$, ISNPRJ and pseudo-random sequence phase-modulation produce lagged four false targets and suppression jamming areas. Non-uniform repeater jamming based on shift frequency modulation and joint modulation interference can coherently accumulate to form a high-amplitude false target near the real target. The interference range is 80 m, the maximum amplitude is as high as 11.3 dB, and the interference distribution is approximated in Figure 13a,b. When $T_{\varphi }=0.1\;$μs, the interference performance of the non-uniform repeater interference based on shift frequency modulation and joint modulation interference is still good. Due to the reduction in the phase modulation code width, the joint modulation interference range is doubled to 160 m, and the maximum interference amplitude is slightly reduced to 8.3 dB. Furthermore, the distribution characteristics of the joint modulation interference are more irregular. Therefore, the interference performance of the joint modulation interference is similar to that of the non-uniform repeater jamming based on shift frequency modulation for LFM signals. Its advantage is that it can broaden the interference range and destroy the interference distribution law by reducing the phase modulation code width.

**Figure 14:**
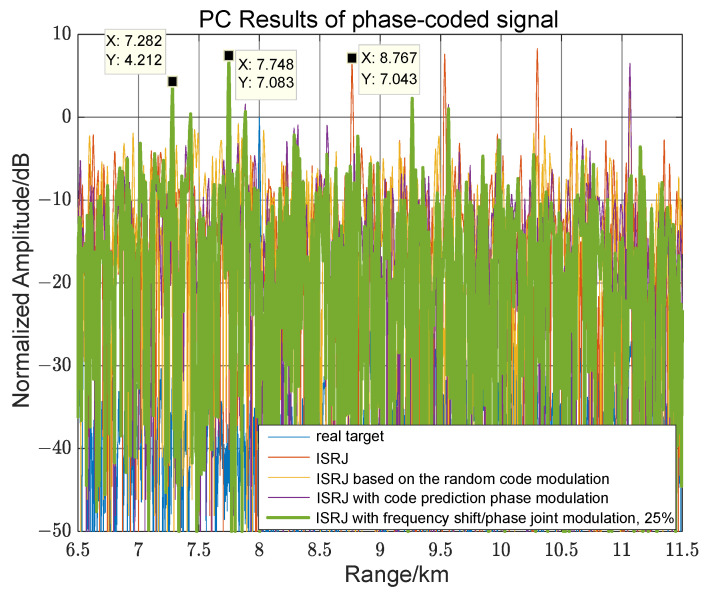
Comparative analysis of joint modulation methods and other methods for phase-coded signals.

Suppose $M_{J}=4$, η=25%, $T_{f}=\tau /2$, and $T_{\varphi }=0.1$ μs, the predicted code sequence interval matrix is γ=[009999119119694900], and the frequency shift matrix ξ=[000000002020000000002020] MHz. Figure 14 depicts the pulse compression results of the joint modulation jamming and other modulation jamming methods for phase-coded signals. Among them, code prediction phase modulation and joint modulation can generate pre-lead false targets. Because $\gamma_{1,0}=\gamma_{1,1}=99$, the pre-lead false target position formed by the first forwarding is $R_{0}+c\cdot \left(\tau -\gamma_{1,0}\cdot T_{\varphi }\right)/2$ = 7.28 km, and the interference amplitude is 4.2 dB. When $\gamma_{2,0}=\gamma_{2,1}=119$, the pre-lead false target position formed by the second forwarding is $R_{0}+c\cdot \left(2\tau -\gamma_{2,0}\cdot T_{\varphi }\right)/2$ = 7.75 km, and the interference amplitude is 7 dB. Because $\xi_{0,8}=\xi_{0,9}=\xi_{1,8}=\xi_{1,9}=20$ MHz, $\gamma_{4,0}=\gamma_{4,1}=0$, the lagging false target formed by the fourth forwarding is eliminated due to the effect of the frequency shift. Therefore, compared with code predictive modulation, joint modulation can adjust the amount of interference through a frequency shift and destroy some unwanted interference.

## 6. Conclusions

In this paper, an ISRJ based on joint frequency shift/phase modulation was proposed to overcome the limitations of ISRJ for LFM and phase-coded signals. Combined with the ISRJ-matched filtering, segmented frequency shift modulation, and phase modulation characteristics for LFM and phase-coded signal, the influence of frequency shift and phase modulation parameters on the amplitude, position, and interference range of the false targets were analyzed. The modulation parameters were flexibly controlled for different signals to achieve good interference performances, such as the formation of the pre-lead false target, irregular spatial distribution characteristics of interference, and increased interference range. The proportion of ISRJ signal participating in frequency shift modulation determines the amplitude of the pre-lead false target. Therefore, amplitude compensation should be used to improve the jamming power and achieve effective jamming. In addition, the combination of the joint frequency shift/phase modulation strategy and time–domain modulation method will also be the focus of research in the future.

## Figures and Tables

**Table 1:** Simulation parameters.

Parameter	Symbol	Unit	Value
Signal-to-noise ratio	SNR	dB	0
Jamming-to-signal ratio	JSR	dB	20
Pulse width	$T_{p}$	μs	51.1
Carrier frequency	$f_{c}$	GHz	3
Sampling frequency	$f_{s}$	MHz	200
Band	B	MHz	50
Target location	$R_{0}$	km	8
Interrupted sampling pulse width	τ	μs	5.11
Interrupted sampling period	$T_{s}$	μs	25.55
The number of interrupted sampling periods	$N_{J}$		2

## Data Availability

Data sharing not applicable.
